# Precise GDP Spatialization and Analysis in Built-Up Area by Combining the NPP-VIIRS-like Dataset and Sentinel-2 Images

**DOI:** 10.3390/s24113405

**Published:** 2024-05-25

**Authors:** Zijun Chen, Wanning Wang, Haolin Zong, Xinyang Yu

**Affiliations:** 1 College of Resources and Environment, Shandong Agricultural University, Tai’an 271018, China; 15653310918@163.com (Z.C.);; 2 Department of Real Estate Appraisal, Royal Agricultural College, Cirencester, Gloucestershire GL7 6JS, UK; 3 Tropical Research and Education Center, University of Florida, Homestead, FL 33031, USA

**Keywords:** GDP spatialization, secondary and tertiary industries, NPP-VIIRS-like dataset, Sentinel-2 images, comprehensive nighttime light index, random forest classification

## Abstract

Spatialization and analysis of the gross domestic product of second and tertiary industries (GDP_23_) can effectively depict the socioeconomic status of regional development. However, existing studies mainly conduct GDP spatialization using nighttime light data; few studies specifically concentrated on the spatialization and analysis of GDP_23_ in a built-up area by combining multi-source remote sensing images. In this study, the NPP-VIIRS-like dataset and Sentinel-2 multi-spectral remote sensing images in six years were combined to precisely spatialize and analyze the variation patterns of the GDP_23_ in the built-up area of Zibo city, China. Sentinel-2 images and the random forest (RF) classification method based on PIE-Engine cloud platform were employed to extract built-up areas, in which the NPP-VIIRS-like dataset and comprehensive nighttime light index were used to indicate the nighttime light magnitudes to construct models to spatialize GDP_23_ and analyze their change patterns during the study period. The results found that (1) the RF classification method can accurately extract the built-up area with an overall accuracy higher than 0.90; the change patterns of built-up areas varied among districts and counties, with Yiyuan county being the only administrative region with an annual expansion rate of more than 1%. (2) The comprehensive nighttime light index is a viable indicator of GDP_23_ in the built-up area; the fitted model exhibited an R^2^ value of 0.82, and the overall relative errors of simulated GDP_23_ and statistical GDP_23_ were below 1%. (3) The year 2018 marked a significant turning point in the trajectory of GDP_23_ development in the study area; in 2018, Zhoucun district had the largest decrease in GDP_23_ at −52.36%. (4) GDP_23_ gradation results found that Zhangdian district exhibited the highest proportion of high GDP_23_ (>9%), while the proportions of low GDP_23_ regions in the remaining seven districts and counties all exceeded 60%. The innovation of this study is that the GDP_23_ in built-up areas were first precisely spatialized and analyzed using the NPP-VIIRS-like dataset and Sentinel-2 images. The findings of this study can serve as references for formulating improved city planning strategies and sustainable development policies.

## 1. Introduction

The secondary industry refers to the industrial sector engaged in the production and processing of physical products, including manufacturing, mining, and construction. The tertiary industry refers to the industrial sector that provides services and consumer activities, such as retail, financial services, catering, etc. The gross domestic product contributed by the secondary and tertiary industries (GDP_23_) serves as a vivid reflection of human social and economic activities, effectively depicting the economic status of regional development [1,2]. However, conventional statistical approaches for GDP lack spatial information and homogenize data within spatial units. Therefore, employing spatial analysis techniques to examine GDP data is an essential method to address these limitations [3,4]. Night light data can provide a more accurate representation of human social and economic activities, particularly in built-up areas dominated by secondary and tertiary industries. Consequently, it is crucial to develop methods for spatializing GDP_23_ in order to facilitate resource allocation and maintain coordinated sustainable development between socioeconomic systems and ecosystems.

Nighttime light (NTL) datasets based on remote sensing technology, such as DMSP-OLS and NPP-VIIRS, have clear advantages compared with the conventional socioeconomic census in GDP spatialization [5,6]. The OLS sensor carried by the DMSP satellite, launched in 1976, is the world’s first nighttime light data sensor and provides the most extended time series of NTL data [7,8]. In 2013, the Earth Observation Group of the National Oceanic and Atmospheric Administration’s National Geophysical Data Center (NOAA/NGDC) released the first global NPP-VIIRS NTL data [9]. As a new generation of data, NPP-VIIRS data provide more excellent spatial resolution and a broader radiometric detection range than the conventional DMSP-OLS NTL data [10,11,12]. For instance, Li et al. used NPP-VIIRS data for flood monitoring and developed software for real-time monitoring [13]. Lv et al. used DMSP-OLS and NPP-VIIRS data combined with China’s CO_2_ emissions and energy consumption data to develop a CO_2_ emission estimation model [14]. Currently, NTL imagery has been widely utilized in social area development research, primary event assessment [15], and human economic activity [16,17,18]. For instance, Goodchild et al. first combined spatial data with socioeconomic data for spatialized analysis [19]. Elvidge et al. used lighting data from 21 countries and confirmed a strong correlation between GDP and nighttime lighting data [20]. Doll et al. used DMSP-OLS data to produce a GDP density map for 11 European countries [21]. Henderson et al. found that night lighting data are a good data source for analyzing long-term GDP growth trends and short-term fluctuations [22]. Weidmann and Schutte used NTL data to estimate GDP in developing countries [23].

The commonly used DMSP-OLS and NPP-VIIRS, however, have limitations such as inconsistent timing, data overflow owing to resolution variances, inherent disparities in detection capability of different sensors, and imaging time differences [24,25]. To address these issues, Chen et al. proposed a self-encoder-based cross-sensor (DMSP-OLS and NPP-VIIRS) NTL data correction scheme and created the first worldwide 500-m resolution annual-scale “NPP-VIIRS-Like” NTL dataset with high data quality comparable to NPP-VIIRS. It can display precise information about the inner city, including annual time series variations in population and light intensity at various scales [26]. More studies using the NPP-VIIRS-like dataset in typical cities are expected to examine its feasibility in urban planning and regional development.

The secondary and tertiary industries of GDP (GDP_23_) play a pivotal role in the GDP contribution of cities [27,28]. NTL data effectively reflect the progress of these industries, yet existing studies often conflate them with the primary industry during analysis, highlighting an urgent need for further investigation. Considering that the activities of secondary and tertiary industries predominantly occur within built-up areas [29,30], accurately extracting built-up areas has emerged as a prominent research focus. Zheng et al. used NPP-VIIRS to monitor the dynamic changes in urban built-up areas [31]. Li et al. [32] and Zhao et al. [33] extracted built-up areas by using multi-period night light data through statistical data comparison method. However, NTL data exhibit greater sensitivity to urban identification at a large scale. Nevertheless, relying solely on night light image data may lead to confusion between construction land and bare land, as well as being influenced by spatial resolution and statistical data, resulting in limited accuracy of extraction. The exploration of effectively leveraging the advantages of the NTL imagery and auxiliary data set to accurately delineate built-up areas and subsequently monitor the spatial characteristics of GDP_23_ has emerged as an imperative issue.

Zibo is a typical industrial city in China, renowned for its robust secondary and tertiary industries. This study thus selected Zibo city as the study area, and NPP-VIIRS-like dataset and multi-temporal Sentinel-2 images as the main study data sources, which were used to extract the night lighting magnitudes in the built-up area, analyze the relationship between nighttime lighting data in the built-up area and GDP_23_, build models to simulate spatialized GDP_23_, and investigate the regional economy’s spatial distribution patterns. The findings are expected to provide a new theoretical and methodical reference for the detailed study of GDP_23_ spatialization in built-up areas and lay a solid foundation for enhanced resource allocation and coordinated development at the regional level.

## 2. Study Area and Data Source

### 2.1. Study Area

**Figure 1:**
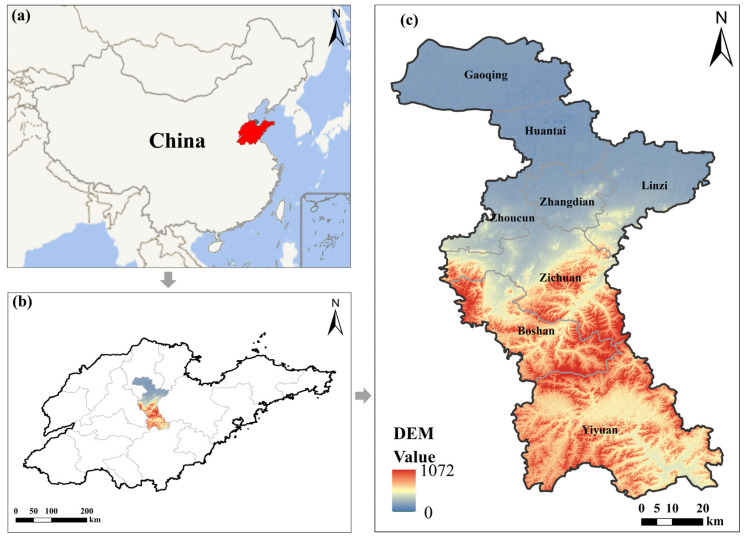
Location of the study area. (**a**) Shandong province in China; (**b**) Location of the study area in Shandong province; (**c**) the DEM of the study area.

Zibo city (Figure 1) is located in the middle section of Shandong province, China (35°55′20″–37°17′14″ N, 117°32′15″–118°31′00″ E), with a total area of 5965 km^2^. It is an essential trading and economic center in east China due to its unique geographical location. The study area has a monsoon climate, which is warm and humid with wet and hot periods. The region’s annual average temperature is 13.2 degrees Celsius, and the annual average precipitation is 615.1 mm. Zichuan district, Zhangdian district, Boshan district, Linzi district, Zhoucun district, and Huantai county, Gaoqing county, and Yiyuan county are the eight administrative districts and counties studied.

### 2.2. Data Sources and Preprocessing

The data sources used in this study are shown in Table 1. The Sentinel-2 multispectral images were obtained using the PIE-Engine remote sensing cloud computing platform, and the 2015–2020 NTL remote sensing data used in this study were downloaded from the Harvard Dataverse. The primary geographic information data (administrative boundary) used in this study were obtained from the National Geomatics Center of China. The socioeconomic statistical data of Zibo city was acquired from the Zibo Municipal Statistics Bureau.

The PIE-Engine platform was employed in this study to search and process the Sentinel-2 images. It is an open product designed to meet the demand for efficient information processing and services resulting from the rapid growth in Earth observation data acquisition capabilities (https://engine.piesat.cn/, accessed on 1 November 2020). It serves as an internet-based engine for remote sensing data processing and services, offering robust data storage, high-performance analysis, and computing capabilities. To ensure image clarity and integrity during synthesis, Sentinel-2 multi-spectral images (13 bands, Table 2) of Zibo City acquired in the summer season from 2015 to 2020 were selected as data sources using the PIE-Engine cloud platform with a filter function (pie.filter()) applied to exclude images with less than 5% cloud cover throughout the year. The images were then cropped using the vector administrative boundary data of the study area to extract multispectral images of the study area.

**Figure 2:**
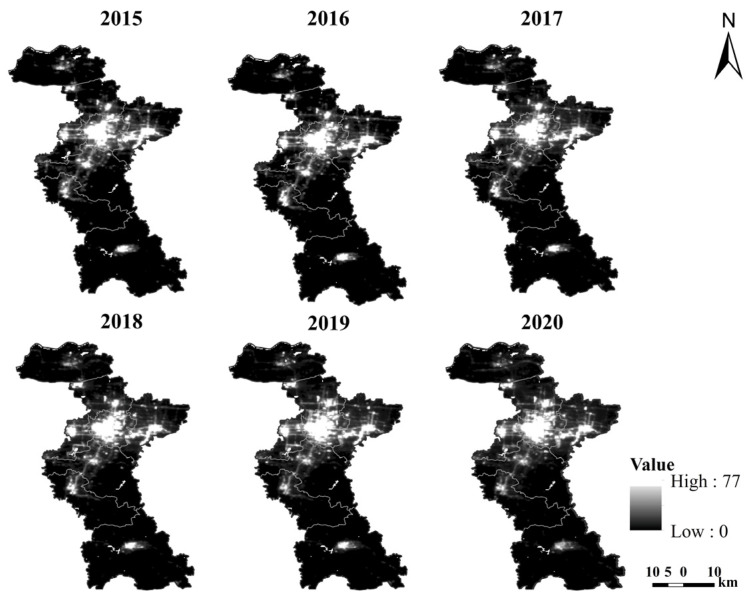
2015–2020 NPP-VIIRS-like nighttime light dataset.

To minimize the projection deformation and improve the accuracy of the study results, the unified data projection of the research image datasets were set to Asia Lambert Conformal Conic, and the data resolution was resampled to 500 m by the cubic convolution resampling method. Using the QGIS extraction and analysis tool, the nighttime light data from 2015 to 2020 of the study area were extracted according to the vector boundary of Zibo city and district/county. The average light intensity, total light intensity, and nighttime light area ratio, which can reflect differences in regional economic conditions, were derived using light data from each county and the city’s overall nighttime light data [34] (Figure 2).

## 3. Methods

**Figure 3:**
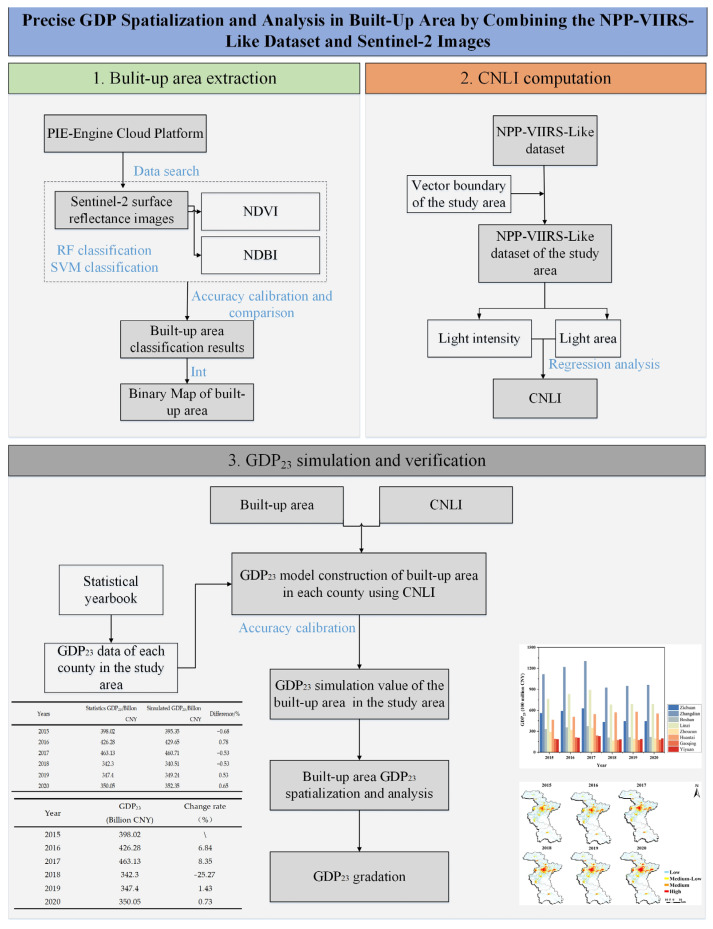
Research workflow.

The research workflow is illustrated in Figure 3. Detailed information is presented below.

### 3.1. Bulit-Up Area Extraction and Comparison with a Conventional Method

Accurate extraction of built-up areas is the key to precise GDP_23_ spatialization. This study selected a random forest (RF) classification model to extract built-up areas. The initial step in constructing an RF classification model involves generating training subsets for each decision tree through the utilization of a random sampling method [35,36]. Subsequently, a subset of the data is employed to train and construct an individual decision tree, with each tree producing one output. Ultimately, the final classification result is determined by aggregating all decision outputs through voting [37]. In this study, sample points were randomly and evenly selected from the PIE-Engine cloud platform, incorporating Sentinel-2 images and high-resolution satellite maps provided by the platform. These sample points were then trained and classified using the pie.Classifier.rTrees() function. Specifically, 70% of the data were utilized as training samples while 30% served as verification samples.

As a typical conventional method approach for classifying multi-spectral images, the support vector machine (SVM) classification method was employed as a comparison in this study to differentiate built-up pixels based on the spectral information of the Sentinel-2 images [38]. The main challenge in automating SVM lies in its supervised nature, requiring users to designate classes of interest and train the classifier prior to classification [39]. In this study, an RBF kernel type was utilized with a gamma value set at 0.5 and a cost parameter of 10.

The user’s and producer’s accuracy were utilized in this study to assess the accuracy of built-up area extraction results generated by the RF and SVM methods. The user’s accuracy indicates the proportion of pixels accurately representing a specific class compared to the total number of pixels. The producer’s accuracy measures how accurately portions of a given class are represented in the categorized image [40]. To further assess the classification results obtained from the RF and SVM classification methods, quantity disagreement and allocation disagreement were introduced to evaluate the classification outcomes during the study period. Quantity disagreement refers to discrepancies between the reference graph and comparison graph due to suboptimal category proportions. Allocation disagreement pertains to disparities in spatial distribution of categories between the reference graph and comparison graph that do not achieve optimal alignment [41]. The formulas for calculating quantity disagreement and allocation disagreement are presented below.

(1) $$q_{g}=\left|\left(\sum_{i=1}^{J}\;p_{ig}\right)-\left(\sum_{j=1}^{J}\;p_{gj}\right)\right|$$

(2) $$Q=\frac{\sum_{g=1}^{J}\;q_{g}}{2}$$

(3) $$a_{g}=2min\left[\left(\sum_{i=1}^{J}\;p_{ig}\right)-p_{gg},\left(\sum_{j=1}^{J}\;p_{gj}\right)-p_{gg}\right]$$

(4) $$A=\frac{\sum_{g=1}^{J}\;a_{g}}{2}$$

where g is the classification type, $q_{g}$ is the quantity disagreement of classification type g, Q is the quantity disagreement of all the classification types, $a_{g}$ is the allocation disagreement of classification type g, and A is the allocation disagreement of all the classification types [42].

Following the accuracy verification, the range of built-up areas generated by the RF classification method was transformed into binary graphs for cropping the computation results generated by the NPP-VIIRS-like dataset.

### 3.2. CNLI Computation

The NPP-VIIRS-like nighttime light data was clipped using the vector boundary of the study area, and then measured using the comprehensive nighttime light index (*CNLI*) method, which takes into account the impact of built-up areas and socioeconomic activities on lighting [42]. It can better portray the relationship between nighttime light and economic activity. The following equations were used to determine the *CNLI*:

(5) CNLI=I×S

(6) $$I=\frac{\sum_{I=P}^{{DN}_{M}}\left({DN}_{i}\times n_{i}\right)}{N_{i}\times {DN}_{M}}$$

(7) $$S=\frac{{Area}_{N}}{Area}$$

where *I* is the average light intensity in an area; *DN_i_* represents the *DN* value of pixels in an area; *n_i_* is the total number of pixels in an area; *DN_m_* is the maximum possible *DN* value in an area; *P* is the threshold for removing invalid value in an area; *N_L_* is the total number of lighting pixels in an area satisfying the condition *DN* value in [*P*,*DN_M_*]; *Area_N_* is the area of lighting pixels in an area meeting the condition *DN* value in [*P*,*DN_M_*]; *Area* is the total area of an area; and *S* is the ratio of the lighting area and total area of the study region.

### 3.3. GDP_23_ Simulation and Verification

To build a viable simulation model, SPSS 20 software was employed here to conduct regression analysis on the data of 8 counties from 2015 to 2020 for GDP_23_ in the built-up area. The following is the GDP_23_ simulation model:

(8) $${GDP}_{23}=a{\left(CNLI\right)}^{2}+b(CNLI)+c$$

where ${GDP}_{23}$ represents the simulated value of GDP_23_, and a, b, c are the coefficients of the regression model.

Following the creation of the GDP_23_ spatial model, the preliminary GDP_23_ simulation value in the built-up areas was calculated using the overall GDP_23_ simulation system. The preliminary simulated GDP data was obtained by setting up fishing nets in the built-up areas, extracting pixel values, and computing pixel values. The error in the simulated GDP_23_ value obtained by simply substituting the value of lighting data was considerable, so the linear correction approach was used to minimize the error. The simulated GDP_23_ value of each pixel in each district/county was adjusted using the GDP_23_ statistical data from the district/county in the study area. The correction equation is shown below.

(9) $${GDP}_{23C}={GDP}_{23i}\times (\frac{{GDP}_{23j}}{{GDP}_{23all}})$$

where ${GDP}_{23C}$ is the corrected simulated GDP_23_ of a pixel, ${GDP}_{23i}$ is the simulated GDP of a pixel, ${GDP}_{23j}$ is the GDP of a district/county, and ${GDP}_{23all}$ is the simulated GDP of a district/county.

An accuracy assessment was performed to study the effect of GDP simulation. The absolute relative error was utilized to evaluate the capacity of the corrected nighttime data to simulate the GDP [29,30]. The relative error was calculated to verify the accuracy using the following equation:

(10) $$\delta =\frac{{GDP}_{23\varepsilon }-{GDP}_{23S}}{{GDP}_{23S}}$$

where ${GDP}_{23\varepsilon }$ is the corrected simulated GDP_23_ of the study area, and ${GDP}_{23S}$ is the statistical ${GDP}_{23S}$ of the study area. Subsequent to the simulation values being corrected by the linear correction method, the simulated GDP_23_ values were allocated to each independent grid, and the spatial inversion and simulation of GDP_23_ were conducted using the QGIS 3.36.2 software. The simulated GDP_23_ layers were then divided into four grades for further analysis: (a) Low GDP_23_ (<20 million CNY/grid), (b) Medium–Low GDP_23_ (20–100 million CNY/grid), (c) Medium GDP_23_ (100–200 million CNY/grid), and (d) High GDP_23_ (>200 million CNY/grid).

## 4. Results

### 4.1. Bulit-Up Area Extraction

The maximum depth of random forest trees and the number of node samples were adjusted in PIE-Engine to validate their impact on accuracy. After comparison, the tree’s maximum depth was set to 20, and the minimum number of node samples was set to 4. Table 3 presents the computed classification accuracies for Sentinel-2 images from 2015 to 2020. In comparison with the SVM classification method, the RF classification method demonstrated an improvement of 16.89% (0.90 vs. 0.77) in extracting built-up areas; additionally, the RF classification method exhibited lower quantity disagreements (4.00–4.50%) compared to those of the SVM classification method (Table 3). It can be concluded that the RF classification method is more suitable for extracting built-up areas in Sentinel-2 images, providing reliable results that can be utilized for further analysis.

**Figure 4:**
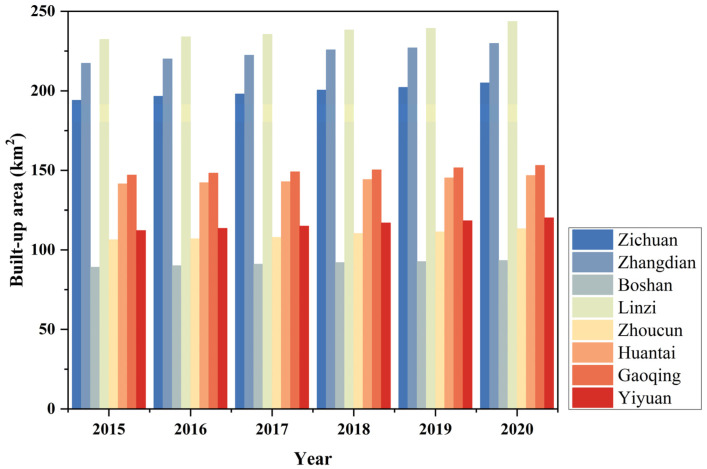
Built-up areas in the study area during the study period.

The built-up areas from 2015 to 2020 in the study area were intersected with the vector boundary data of each district/county, and their areas were computed and presented in Figure 4. The change pattern of built-up area varied among districts and counties. In 2015, Linzi district had the highest extent of built-up area, reaching 232.36 km^2^, whereas Boshan district had the lowest extent, with only 89.12 km^2^. Subsequently, all of the eight districts and counties experienced an increase in built-up area throughout the study period. In 2016, Zichuan, Zhangdian, Boshan, and Yiyuan exhibited change rates exceeding 1%, reaching 196.60, 220.08, 90.08, and 113.59 km^2^; however, in 2017, only Zhangdian, Boshan, and Yiyuan continued to show increasing trends above this threshold. During this period, Huantai county had the slowest expansion rate of built-up area. By 2018, Zhoucun district recorded a significant rise in its built-up area change rate at over 2% (110.33 km^2^, 2.25%), which was the highest among all studied areas during this period. In 2019, the expansion trend of built-up areas weakened, with only Yiyuan maintaining an increasing rate of 1.21%, while the remaining seven districts and counties displayed rates below that level. In 2020, except for Boshan district, the increasing rates of all of the other seven districts and counties exceeded 1%. Among the eight districts/counties, Yiyuan county was the only district/county with an annual expansion rate of more than 1% from 2015 to 2020.

### 4.2. Modeling Performance of GDP_23_ Simulation

The *CNLI* of the built-up areas in the study area from 2015 to 2020 was estimated using Equations (2)–(4). The coefficient of correlation between *CNLI* and GDP_23_ was obtained. The results found that the GDP_23_ and *CNLI* have a correlation coefficient of 0.906. Using Equation (5), the regression model of GDP_23_ and *CNLI* was established as follows:

(11) $${GDP}_{23}=-30083.93\;{\left(CNLI\right)}^{2}+13690.98\;CNLI-462.74$$

**Figure 5:**
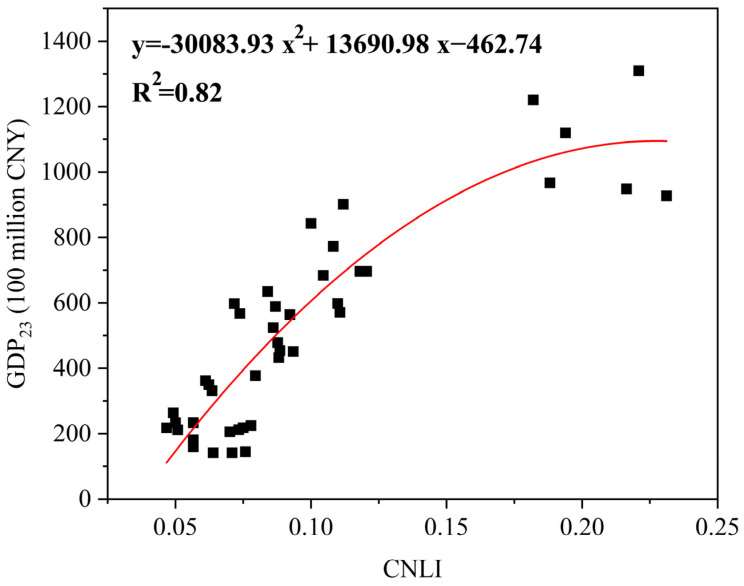
Agreement relationships of GDP_23_ and *CNLI*.

The regression modeling results are shown in Figure 5, which indicates that GDP_23_ and *CNLI* were highly correlated in the study area. GDP_23_ regression models had R^2^ values of 0.82, suggesting that the model built based on the night light index and GDP_23_ data can describe the city’s economic development, and that night light data in the built-up area can be utilized to simulate Zibo city’s GDP_23_ spatial distribution. We found that the overall relative errors of simulated GDP_23_ and statistical GDP_23_ were all below 1% (Table 4), and that the *CNLI* can simulate the study area’s GDP_23_ with high accuracy.

### 4.3. GDP_23_ Spatialization and Analysis in the Study Area

Using the model building for simulated GDP_23_ in the study area, the total GDP_23_ and its annual growth rate were computed and are presented in Table 5. The total GDP_23_ of the study area experienced a steady increase from 2015 to 2017, with figures reaching 398.02 billion China Yuan (CNY), 426.28 billion CNY, and 463.13 billion CNY, respectively, as indicated in Table 5. Similarly, the per capita GDP_23_ followed a similar upward trend over this period, gradually rising to 89,235 CNY, 94,587 CNY, and finally reaching 101,781 CNY, respectively. However, starting from the year 2018 onwards, there was a significant decline in the study area’s GDP_23_ data by more than a quarter (−25.27%). Subsequently, though, after that year’s downturns, there was an eventual recovery where both total GDP_23_ and per capita GDP_23_ increased again, albeit at much slower rates (1.43% and 0.73%) compared to those observed prior to the year of reference (Table 5).

**Figure 6:**
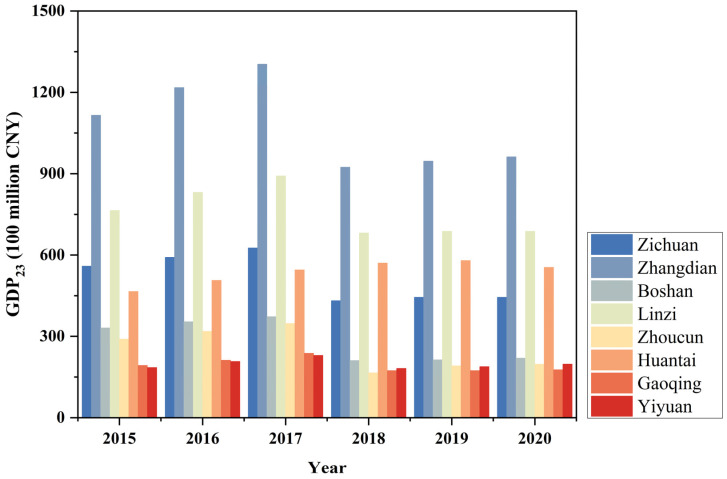
GDP_23_ of each county during the study period.

Figure 6 depicts the GDP_23_ changes in each district/county of the study area. The year 2018 marked a significant turning point in the trajectory of GDP_23_ development in the study area. From 2015 to 2017, all eight districts/counties experienced an upward trend in GDP_23_. Yiyuan county exhibited the highest growth rate with a 12.06% increase in 2016 and a further increase of 10.76% in 2017. In 2018, except for Huantai county (which saw a modest increase of 4.69%), all of the other seven districts/counties witnessed significant declines in their GDP_23_ values. Among them, Zhoucun district, in 2018, had the largest decrease at −52.36%, marking the most substantial decline among them all. Subsequently, the GDP_23_ values gradually recovered across all eight districts/counties in the years 2019 and 2020. Overall, GDP_23_ in the eight districts/counties showed distinct change patterns.

### 4.4. GDP_23_ Gradation

**Figure 7:**
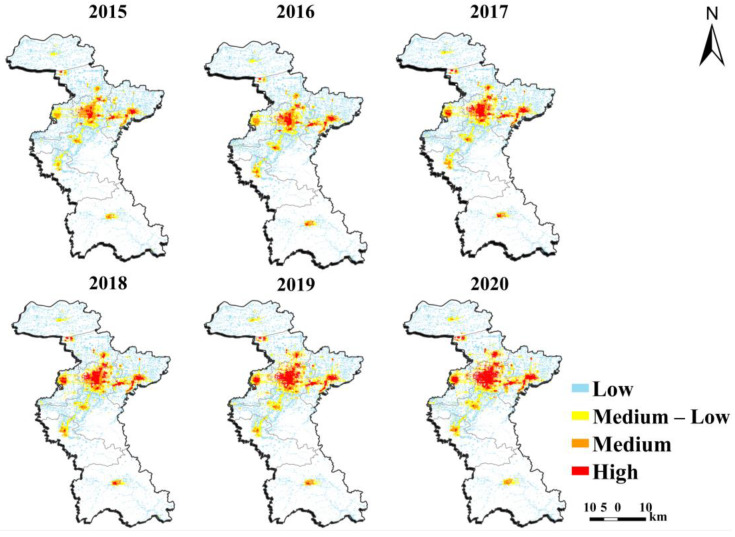
GDP_23_ magnitudes of the study area from 2015 to 2020.

Based on the validated GDP_23_ simulation process, the GDP_23_ density maps of the built-up areas in the study area from 2015 to 2020 were generated and are depicted in Figure 7. Analysis found that there was an imbalanced distribution and disparities in GDP_23_ allocation among districts/counties within the study area. The central region of the study area exhibited a better economic condition with a more symmetrical distribution, and there is obvious inter-annual diffusion during the study period. High GDP_23_ districts were mainly concentrated in the administrative center of Zhangdian district; in addition, Linzi district and Huantai county also have obviously high value areas. The urban areas of Gaoqing county in the northern region and Boshan district and Yiyuan county in the southern region primarily consist of regions with medium–low and low GDP_23_ levels. Overall, as distance increased from the central area, a median area emerged outside it, followed by middle–low value areas and low-value areas, while maintaining a significant dispersion pattern, indicating a gradual diffusion trend from center to periphery.

Table 6 presents the spatialized GDP_23_ statistics for different grades in the districts and counties. The level of GDP_23_ development in the study area was relatively low. As the political, economic, and cultural center of the study area, Zhangdian district exhibits the highest proportion of high GDP_23_, accounting for 9.36%, 14.65%, 23.93%, 28.71%, 34.79%, and 38.18% from 2015 to 2020, respectively, with a consistent year-on-year increase during the study period (Table 6). Driven by Zhangdian district’s growth, Linzi district, Zhoucun district, and Huantai county also witnessed an annual increase in areas with high GDP_23_ (Table 6). Notably, Zhoucun district experienced significant expansion in its high GDP_23_ area, starting from zero in 2015 and reaching a proportion of 11.71% in 2020.

The proportion of regions with medium GDP_23_ in Zhangdian district remains the highest (>30%), while the areas of each district and county fluctuate during the study period. In 2015–2017, the proportion of regions with medium–low GDP_23_ in Zhangdian district was the highest (>27%), whereas after 2018, Zhoucun district had the highest proportion (>28%). Over time, there has been a gradual decrease in the range of regions with medium–low GDP_23_ in Zhangdian district, while Zichuan district and Gaoqing county have experienced an increase year by year. The remaining districts and counties have shown fluctuations.

In addition to the Zhangdian district, the remaining seven districts and counties collectively accounted for over 60% of the low GDP_23_ region. Throughout the study period, with the exception of Boshan district and Linzi district, there was a consistent year-on-year decline in low GDP_23_ areas across the other six districts and counties. Notably, Huantai county experienced a significant decrease of 26%, while Zhoucun district saw a reduction of 23.3% and Zhangdian district witnessed an 18.5% decrease (Table 6).

## 5. Discussions

Spatializing and quantitative analyzing of GDP_23_ are essential for depicting the socioeconomic status of regional development. This study initially combined NPP-VIIRS-like dataset and Sentinel-2 images as the primary data sources and selected Zibo city as the study area for conducting spatial modeling and analysis of GDP_23_ in the built-up area. The main findings revealed that the combination of NPP-VIIRS-like dataset and Sentinel-2 images can precisely depict GDP_23_ in the built-up area, that *CNLI* is a viable indicator with a high fitted model R^2^ of 0.82, and that the overall relative errors of simulated GDP_23_ and statistical GDP_23_ were below 1%. The year 2018 was a significant turning point in the trajectory of GDP_23_ development in the study area; the level of GDP_23_ development in the study area was relative low, and the patterns of GDP_23_ grades varied among the eight districts/counties. The findings of this study can serve as valuable references for city planning and sustainable development.

The spatialization analysis results found that the GDP_23_ was unevenly distributed in the study area, which may be related to the urban functions of the study area and the policies of the government implemented during the study period. The spatial distribution of GDP_23_ is influenced by urban function [43]. Figure 7 shows that high GDP_23_ areas were concentrated in Zhangdian district, the municipal administrative center of Zibo city, from which radiated Linzi district, Zichuan district, and Huantai county, with a scattered distribution in the surrounding areas. The key industrial areas of the study area were these four districts and counties. They were Zibo city’s economic core area and supported the city’s main economic development. Conversely, there are many mountainous areas in Boshan district, Gaoqing county, and Yiyuan county, with a large proportion of agricultural land and primary industry among industrial types, which were the main ecological and environmental protection areas, resulting in a large proportion of areas with low GDP_23_ in these built-up areas.

Furthermore, policy is a significant driving factor in the changing of GDP’s spatial features [44,45,46]. Table 5 indicated that there was a significant decline in the study area’s GDP_23_ data by more than 25% in 2018. China implemented new environmental protection policies in 2017 to ensure sustainable development. In the year 2017, following this policy, the Zibo government formulated coping strategies and initiated the transformation of both established and emerging driving forces [47]. Many heavy chemical firms in the Zhangdian district have begun to pursue transformation and upgrading to change the economic development model, slow the rate of economic growth, and improve economic quality [48,49]. In 2018, the study area closed hundreds of low-efficiency and high-pollution enterprises and focused on industrial agglomeration. As shown in Figure 7, the GDP_23_ density center shifted northward, settling in the Zhangdian district and Huantai county. Even though the industrial belt in Huantai county was declining, the overall trend in Huantai county, as a key industrial producing area in the study area, was generally stable. The reduction in GDP_23_ density in Yiyuan county and Boshan district is depicted in Figure 7. Yiyuan county and Boshan district were transferring traditional industries rationally, continuously improving environmental quality, carrying out ecological environment protection with high standards, and paying more attention to natural resource protection and industrial utilization transformation to meet the requirements of environmental protection construction.

Figure 5 showed that the study area was mostly concentrated in the range of less than 0.1 and larger than 0.16, with a gap between them in the simulation modeling findings, indicating that the study area lacks middle-level economic counties. This occurrence may be connected to Zibo’s urban structure. The study area is a representative group city [43]; distinct districts and counties serve different purposes, and there is little cohesiveness and driving force among them. Overall, Zhangdian district, Linzi district, and Huantai county drove the economic development of the study area. The lack of transitional medium-sized economic counties led to the uneven distribution of urban nighttime light.

To facilitate the advancement of medium- and high-sized economic counties, Zibo City should persist in promoting the transition of traditional industries into high-tech and environmentally sustainable sectors, fostering green and low-carbon development to enhance the quality of the ecological environment, as well as reinforcing natural resource conservation. The government should actively promote the development of the environmental protection industry, with a particular focus on enhancing efforts in environmental governance, manufacturing of environmental protection equipment, and comprehensive utilization of industrial resources. This includes prioritizing the production of non-toxic denitrification catalysts, advancing the manufacturing of environmental protection equipment (such as air pollution control systems, water treatment facilities, solid waste treatment technologies), and promoting the comprehensive utilization of recycled resources and fly ash to establish a complete industrial chain. Additionally, the government can actively promote the development of high-tech industries by strategically investing in emerging sectors such as intelligent connected vehicles, hydrogen energy, digital economy, and smart logistics. This will serve as a catalyst for industrial innovation and facilitate its upgrading.

The findings of this study indicate that the combination of NPP-VIIRS-like dataset and Sentinel-2 images is more effective in capturing social and economic conditions at the county level; however, this study has limitations. Firstly, there have been studies using MANet [50] and MLNet [51] deep learning methods for the extraction of land use types. Further study can be conducted to explore the feasibility of these methods to identify built-up areas. Secondly, this study did not examine the feasibility of town-level application. Considering the spatial resolution of the NPP-VIIRS-like dataset used in the study, the study may primarily be suitable for regional research at or above the county level. Thirdly, this study used the NPP-VIIRS-like dataset and Sentinel-2 images to investigate the socioeconomic conditions in the built-up area of Zibo city. Future studies will examine this framework in other regions to further explore their feasibility and integrate nighttime light data with other socioeconomic indicators for a more comprehensive analysis of socioeconomic dynamics and urban structural transformations, thereby expanding the application scope of nighttime light datasets.

## 6. Conclusions

This study investigated the feasibility of combining the NPP-VIIRS-like dataset and Sentinel-2 images to precisely spatialize and analyze the variation patterns of the GDP_23_ in a typical city’s built-up area. The results found that the RF classification method can accurately extract built-up area with satisfied accuracies and low disagreements; the change patterns of built-up area varied among districts and counties; Yiyuan county was the only administrative region with an annual expansion rate of more than 1%. The *CNLI* is a viable indicator of GDP_23_ in the built-up area; the fitted model exhibited an R^2^ value of 0.82, and the overall relative errors of simulated GDP_23_ and statistical GDP_23_ were both below 1%. The year of 2018 was a significant turning point in the trajectory of GDP_23_ development in the study area; in 2018, Zhoucun district had the largest decrease in GDP_23_ at −52.36%. GDP_23_ gradation found that the level of GDP_23_ development in the study area was relatively low. Zhangdian district exhibits the highest proportion of high GDP_23_ (>9%); all the remaining seven districts and counties collectively accounted for over 60% of the low GDP_23_ region. This study first precisely spatialized and analyzed the GDP_23_ in built-up area by combining the NPP-VIIRS-like dataset and Sentinel-2 images; the findings can serve as valuable references for formulating improved city planning strategies and sustainable development policies.

## Figures and Tables

**Table 1:** Data source of this study.

Data	Period	Source
Sentinel-2 multispectral image	2015–2020	PIE-Engine platform (https://engine.piesat.cn, accessed on 1 November 2020)
NTL dataset	2015–2020	Harvard Dataverse (https://dataverse.harvard.edu/dataset.xhtml?persistentId=doi:10.7910/DVN/YGIVCD, accessed on 1 January 2021)
Administrative boundary	2020	National Geomatics Center of China (http://www.ngcc.cn, accessed on 28 December 1995)
Socioeconomic statistical data	2016–2021	Zibo Municipal Statistics Bureau (http://tj.zibo.gov.cn, accessed on 1 June 1980)

**Table 2:** Band information of Sentinel-2 multispectral camera sensor.

ID	Band	Center Wavelength (nm)	Resolution (m)
1	Band 1-Coastal aerosol	0.443	60
2	Band 2-Blue	0.49	10
3	Band 3-Green	0.56	10
4	Band 4-Red	0.665	10
5	Band 5-Vegetation Red Edge	0.705	20
6	Band 6-Vegetation Red Edge	0.74	20
7	Band 7-Vegetation Red Edge	0.783	20
8	Band 8-NIR	0.842	10
9	Band 8A-Vegetation Red Edge	0.865	20
10	Band 9-Water vapor	0.945	60
11	Band 10-SWIR-Cirrus	1.375	60
12	Band 11-SWIR	1.61	20
13	Band 12-SWIR	2.19	20

**Table 3:** Accuracy of built-up area extraction using the RF and SVM classification method.

Accuracy Assessment Type	Classification Method	
	RF	SVM
User’s Accuracy	0.91–0.93	0.78–0.82
Producer’s Accuracy	0.90–0.92	0.77–0.79
Quantity Disagreement (%)	4.00–4.50	14.50–15.75
Allocation Disagreement (%)	11.25–11.50	5.25–5.90

**Table 4:** Verification of the accuracy of simulated GDP_23_ in the study area.

Years	Statistics GDP_23_/Billon CNY	Simulated GDP_23_/Billon CNY	Difference/%
2015	398.02	395.35	−0.68
2016	426.28	429.65	0.78
2017	463.13	460.71	−0.53
2018	342.3	340.51	−0.53
2019	347.4	349.24	0.53
2020	350.05	352.35	0.65

**Table 5:** GDP_23_ statistics of the study area.

Year	GDP_23_ (Billion CNY)	Change Rate (%)
2015	398.02	\
2016	426.28	6.84
2017	463.13	8.35
2018	342.3	−25.27
2019	347.4	1.43
2020	350.05	0.73

**Table 6:** Gradation of simulated GDP_23_ in the study area.

	Zichuan	Zhangdian	Boshan	Linzi	Zhoucun	Huantai	Gaoqing	Yiyuan
2015								
High GDP_23_ (%)	0.43	3.72	0.76	1.35	0.00	1.74	0.03	0.33
Medium GDP_23_ (%)	1.98	24.47	2.33	6.91	3.67	4.15	0.90	0.91
Medium–low GDP_23_ (%)	15.52	61.57	15.57	30.23	33.05	18.29	4.90	3.95
Low GDP_23_ (%)	82.07	10.24	81.33	61.51	63.27	75.82	94.17	94.80
2016								
High GDP_23_ (%)	0.64	6.96	1.18	2.49	0.23	2.37	0.15	0.65
Medium GDP_23_ (%)	2.08	25.02	3.08	7.43	5.35	4.16	1.20	0.84
Medium–low GDP_23_ (%)	13.85	56.25	14.17	25.92	30.89	20.24	11.83	3.72
Low GDP_23_ (%)	83.43	12.62	81.57	64.17	63.53	73.22	86.82	94.79
2017								
High GDP_23_ (%)	0.38	5.22	0.73	2.05	2.08	1.94	0.06	0.4
Medium GDP_23_ (%)	1.4	26.56	0.42	6.08	4.43	3.68	0.48	0.69
Medium–low GDP_23_ (%)	16.65	50.17	1	18.12	24.24	15.5	3.91	2.31
Low GDP_23_ (%)	81.58	18.06	97.85	73.76	69.25	78.89	95.55	96.6
2018								
High GDP_23_ (%)	0.00	0.60	0.03	1.09	2.00	2.27	0.03	0.35
Medium GDP_23_ (%)	1.40	21.63	1.95	5.84	4.20	5.07	0.18	0.78
Medium–low GDP_23_ (%)	17.90	60.21	6.23	20.02	20.78	29.72	7.49	2.54
Low GDP_23_ (%)	80.68	17.56	88.09	73.05	73.02	62.93	92.30	96.33
2019								
High GDP_23_ (%)	0.00	0.47	0.00	0.91	2.40	1.55	0.00	0.15
Medium GDP_23_ (%)	1.40	20.83	1.39	5.58	5.09	5.12	0.30	1.14
Medium–low GDP_23_ (%)	20.62	60.41	10.20	27.79	26.51	27.69	4.87	2.48
Low GDP_23_ (%)	77.98	18.29	88.37	65.72	66.00	65.64	94.60	96.23
2020								
High GDP_23_ (%)	0.00	0.53	0.00	0.88	3.00	1.11	0.05	0.14
Medium GDP_23_ (%)	0.87	22.03	1.36	5.25	5.90	4.78	0.15	1.16
Medium–low GDP_23_ (%)	23.04	63.35	12.29	32.06	29.87	32.09	6.39	2.89
Low GDP (GDP_23_)	76.07	14.09	86.35	61.82	61.08	62.01	93.41	95.82

## Data Availability

All data generated or analyzed during this study are included in this article.
